# Notes in Practice

**Published:** 1877-04-20

**Authors:** C. L. Gregory

**Affiliations:** North Star, O.


### NOTES IN PRACTICE.

By C. L. GREGORY, M.D., North Star, O.

Sciatic and crural neuralgia usually yield promptly to half teaspoonful doses of a strong tinct. gum guaiac every three hours, in conjunction with 15 to 20 gr. doses of blue mass every alternate night. Enough mass must be given to clear the bowls rather freely. An excellent prescription is the following:

R—Fl. Ext. Gelseminum......
" " Black Cohosh..a a oz. i M.
S. Ten drops every four hours until the toxical effect of the gelseminum, drooping of the eye-lids, is noticed, when the dose should gradually be diminished.

In severe cases of malarial and continued fever an agonizing pain often attacks the shoulder, elbow, hip, knee, etc., and becomes almost unbearable. I saturate a woolen cloth with chloroform and apply to the painful part, covering it with a dry cloth to prevent too rapid evaporation. This gives prompt relief. Internally I give:

R—Fl. Ext. Gelseminum.......
" " Black Cohosh, a a oz. i M.
S. Five to ten drops every three or four hours, as above.

I use the above prescription when the limb is left stiff and painful during and after convalescence.

My partner, Dr. A. Pearson, cured an old case of neuralgic pain of the hip, which had been diagnosed morbus coxarius by another surgeon, with the above named prescription. Menstrual suppression from cold and exposure was present in this case, but the menses became regular as the neuralgia was cured. She could bear no weight on the affected limb. No opiates were used after the first week of treatment. Time, eight weeks. In many neuralgias, but especially trifacial, I almost invariably prescribe gelseminum. There are but few cases of dental neuralgia that it will not promptly relieve. In this malarious district quinine is also an excellent remedy. Of course the general health must be attended to.

I have been very successful in curing pain in the kidney, spermatic cord, and testicle with:

R—Bal. Copaiva ..............dr. ii
Sweet Spts. Nit...........oz. ii M.
S. One c. p. every four hours.

Keep the bowels regular by small doses of rhei and aloes, but do not purge.

Some women—and men too—are subject to pain in region of heart, with palpitation and shortness of breath, etc. I prescribe:

R—Fl. Ext. Valerian........ oz, i
Chloroform................ dr. i
Alcohol..................... dr. vii M.

S. One-half to one c. p. every 15 to 30 minutes 'til relieved, then as indicated. Dilute it well with water or syrup.

It acts exceedingly well in those cases, and also in hysterical attacks. I use it in some cases of labor, especially when the woman is anxious, nervous, and excited; also when the pains seem to be located mostly in the back. It calms her down and labor progresses much more satisfactorily to all concerned.

For dyspepsia and indigestion I prescribe the following:

R—Rhei Pulv.
Cubebs pulv.
Hydras. can. pulv.
Brom. Pot............a a one part
Quinia Sulph........ half part M.

Triturate them thoroughly, and to an adult give twelve gr. doses four times per day.

Mr. C. had a distressing pain in his stomach for which he took a large swallow of spts. camphor, which was pure alcohol saturated with the gum, and as a consequence his mouth, throat, œsophagus and stomach became so inflamed and tender that he could scarcely swallow even liquid food. Various remedies were tried for a week, when I put him on a saturated solution brom. pot., a tablespoonful every three hours. He was completely cured in 48 hours. This also acts well in acute conjunctivitis, one or two drops in the eye four times a day. Used as an injection in simple vaginitis it is good.

In some cases of atonic diarrhea, where the bowels move every time any thing is swallowed, and medicine does no good, I have used a strong decoction of coffee with excellent success. Use it cold and clear, letting an adult drink from a half to one pint every three or four hours. After twelve to twenty-four hours let patient commence on boiled or thickened milk, gradually expanding his bill of fare. Brandy made quite thick with flour is also good in these cases.

In dysmenorrhea a pill composed of one gr. opium to one-half gr. ext. belladonna every three hours is quite beneficial. In the neuralgic form I use, in addition to the pill, gelseminum and cohosh a a, mix, ten drops every two hours. In the neuralgic form it is imperative to institute a tonic, and building up treatment during the inter-catamenial period.

PITYRIASIS VERSICOLOR can be quickly and permanently cured with:

R—Nat. Sulphite..............dr. i
Glycerine...................dr. iv
Water.......................dr. iv M.

Use as a lotion once a day.

Acne in all its forms, except perhaps A. Rasacea, can be cured by restraining the appetite, eating very moderately and having the diet constituted principally of oily articles. Also use the following after meals:

R--Solut. potassa arsen.
S. Two to six drops in water.

